# A Single Oral Administration of Theaflavins Increases Energy Expenditure and the Expression of Metabolic Genes

**DOI:** 10.1371/journal.pone.0137809

**Published:** 2015-09-16

**Authors:** Naoto Kudo, Yasunori Arai, Yoshitomo Suhara, Takeshi Ishii, Tsutomu Nakayama, Naomi Osakabe

**Affiliations:** 1 Department of Bio-science and Engineering, Shibaura Institute of Technology, 307 Fukasaku, Munumaku, Saitama, 337–8570, Japan; 2 Department of Food and Nutritional Sciences, University of Shizuoka, Suruga-ku, Shizuoka 422–8526, Japan; 3 School of Food Science and Technology, Nippon Veterinary and Life Science University, Musashinoshi, Tokyo, 180–8602, Japan; Sanford-Burnham Medical Research Institute, UNITED STATES

## Abstract

Theaflavins are polyphenols found in black tea, whose physiological activities are not well understood. This study on mice evaluated the influence of a single oral administration of theaflavins on energy metabolism by monitoring the initial metabolic changess in skeletal muscle and brown adipose tissue (BAT). Oxygen consumption (VO_2_) and energy expenditure (EE) were increased significantly in mice treated with theaflavin rich fraction (TF) compared with the group administered vehicle alone. There was no difference in locomotor activity. Fasting mice were euthanized under anesthesia before and 2 and 5, 20-hr after treatment with TF or vehicle. The mRNA levels of uncoupling protein-1 (UCP-1) and peroxisome proliferator-activated receptor gamma coactivator-1α (PGC-1α) in BAT were increased significantly 2-hr after administration ofTF. The levels of UCP-3 and PGC-1α in the gastrocnemius muscle were increased significantly 2 and 5-hr after administration of TF. The concentration of phosphorylated AMP-activated protein kinase (AMPK) 1α was also increased significantly in the gastrocnemius 2 and 5-hr after treatment with TF. These results indicate that TF significantly enhances systemic energy expenditure, as evidenced by an increase in expression of metabolic genes.

## Introduction

Theaflavins are a type of polyphenols that are present in high concentrations in black tea. Four major theaflavins, theaflavin (TF1), theaflavin-3-O-gallate (TF2A), theaflavin 3’-O-gallate (TF2B), and theaflavin-3,3’-di-O-gallate (TF3), are formed from co-oxidation of selected pairs of green tea catechins during fermentation [1,2]. These catechins include (-)-epicatechin, epigallocatechin, epicatechin-3-gallate, and epigallocatechin 3-gallate. During the processing of black tea, fresh leaves are crushed and allowed to undergo oxidation by polyphenol oxidase, resulting in the formation of polyphenolic oligomers known as theaflavins [2]. Previous surveys have reported a constant level of theaflavin consumption in European countries, especially the UK [3, 4], and in the USA [5] and Japan [6]. In addition, several meta-analyses have shown that consumption of black tea results in significant primary prevention of cardiovascular diseases by decreasing plasma LDL cholesterol levels and blood pressure [7, 8]. It has also been reported that consumption of black tea is inversely associated with body mass index [5]. The biological activity of green tea and its catechins have been studied extensively, with numerous reports suggesting that green tea catechins prevent obesity [9], cardiovascular disorders [10], and cancer [11]. While it is known that black tea represents 78% of tea production worldwide [12], there has been only limited research on the biological significance of ingestion of theaflavins. In the present study in mice, we examined the effect on energy expenditure of administering a single oral dose of theaflavin rich fraction t (10 mg/kg), using indirect calorimetry and monitoring the initial metabolic changes in skeletal muscle and BAT.

## Materials and Methods

### Materials

Crude theaflavin, prepared by incubating tea catechins in the presence of polyphenol peroxidase, was purchased from Yaizu Suisankagaku- Industry Co. Ltd., Shizuoka, Japan. The purified theaflavin mixture was prepared as follows. Catechins and caffeine in the crude theaflavin were excluded using medium pressure column chromatography with a reverse-phase preparative column and a gradient mobile phase of 10 to 80% acetonitrile. The resulting mixture was composed of TF1, TF2A, TF2B, and TF3. The concentration of each theaflavin in this theaflavin rich fraction(TF) was analyzed by HPLC [13] and shown to be: TF1, 4.6%; TF2A, 16.6%; TF2B, 11.2%; and TF3, 26.2%.

### Animals and ethics

This study was approved by the Animal Care and Use Committee of the Shibaura Institute of Technology (Permit Number: 27–2956). All animals received humane care under the guidelines of this institution. Male ICR mice weighing 35–40 g (8–10 wks) were obtained from Charles River Laboratories Japan, Inc. (Tokyo, Japan). The mice were kept in a room with controlled lighting (12/12-hr light/dark cycles) at a regulated temperature between 23–25°C. A certified rodent diet was obtained from the Oriental Yeast Co., Ltd., Tokyo, Japan.

### Experimental procedures

After 4-d on a basal diet (containing 5.0% fat), the animals were divided into 2 groups; vehicle group (n = 8) administered 4 ml/kg distilled water orally and TF group (n = 8) administered 10 mg/kg TF orally. Mice were adapted in single plastic cage 2 days before the measurement. Each animal was placed inside an open-circuit metabolic chamber for a 20-hr period of fasting. VO_2_ and excreted VCO_2_ were determined using a small animal metabolic measurement system (MK-5000RQ Muromachi Kikai Co. Ltd, Tokyo, Japan). The system monitored VO_2_ and VCO_2_ at 3-min intervals and calculated the RER (RER = VCO_2_/ VO_2_). The VO_2_ and VCO_2_ measurements were converted to EE (kcal/ 20-hr, 8-hr of light cycle or 12-hr of dark cycle) using the Weir equation: EE = (3.941 VO_2_ + 1.11 VCO_2_) * 1.44 * 60 min * hr. To measure spontaneous motor activity whilst sedentary, the mice were placed one at a time in a chamber equipped with an infrared-ray passive sensor system (MMP10, Muromachi Kikai). This experiment involved measurements during the dark (18:00–6:00) and light periods (12:00–18:00 and 6:00–8:00). The next experiment involved 8 animals per group being euthanized under pentobarbital anesthesia (50 mg/kg body weight IP, Tokyo Chemical Industry, Tokyo, Japan), before and 2, 5, and 20 hr after administration of their group’s treatment without provision of further food. Tissue samples were collected by dissection and snap frozen in liquid nitrogen and stored at -80°C until analysis.

### Quantitative RT-PCR analysis

Total RNA was prepared from the gastrocnemius muscle and BAT using TRIzol reagent (Life Technologies, California, USA) according to the manufacturer’s instructions. Briefly, 10 μg of total RNA was reverse-transcribed in a 20 μl reaction with high capacity cDNA Reverse Transcription kits (Applied Biosystems, California, USA,). Real-time reverse-transcription (RT)-PCR, using100 ng of total cDNA, was carried out using the StepOne Real-Time PCR System (Applied Biosystems). Primer and probe sequences were selected using a Taqman Gene Expression Assay (Applied Biosystems, S1 Table) and included the following gene and catalog numbers (Applied Biosystems): GAPDH,Mm99999915_g1; UCP-1,Mm_01244861_m1; and UCP-3,Mm_00494077_m1; and PGC-1α,Mm01208835_m1. Glyceraldehyde-3-phosphate dehydrogenase (GAPDH) was used as the internal control. The buffer used in the systems was THUNDER BIRD Prove qPCR Mix (TOYOBO, Tokyo, Japan). The PCR cycling conditions were 95°C for 1 min, followed by 40 cycles at 95°C for 15 s and 60°C for 1 min.

### Western blotting analysis

Tissues were homogenized in a microtube with lysis buffer (CelLytic^TM^ MT cell lysis reagent; Sigma Aldrich, Japan) containing a protease inhibitor (Sigma Aldrich, Japan) and 0.2% SDS. The protein concentration was measured by the Bradford method. Protein (10 μg) was separated by SDS-PAGE using a 4–12% Bis-Tris gel and transferred onto a polyvinylidene difluoride membrane (Life Technology). The membrane was blocked with membrane-blocking reagent (GE Healthcare, Buckinghamshire, UK) for 1 hr. After blocking, the membrane was incubated for 2-hr with a rabbit polyclonal primary antibody against either AMPK1α (1:1600; sc-25792, Santa Cruz Biotechnology, Inc., Santa Cruz,USA), or phosphorylated AMPK1α (1:200; sc-33524, Santa Cruz Biotechnology, Inc., USA). After the primary antibody reaction, the membrane was incubated for 1 hr with appropriate horseradish peroxidase-conjugated secondary antibodies (1:100000). Immunoreactivity was detected by chemiluminescence using the ECL Select Western Blotting Reagent (GE Healthcare). The images of the fluorescence bands were analyzed using Just TLC (SWEDAY, Larkgatan, Sweden) analysis software. The values of phosphorylated-AMPK1α were normalized to those for AMPK1α.

### Data analysis and statistical methods

All data were reported as mean ± standard deviation. Statistical analyses were performed by two or one way ANCOVA, post hoc comparisons with the vehicle group were made by the two-tailed followed by Dunnett's test. A probability of P < 0.05 was considered to be statistically significant.

## Results

### Oxygen consumption resting energy expenditure and locomotor activity

**Fig 1:**
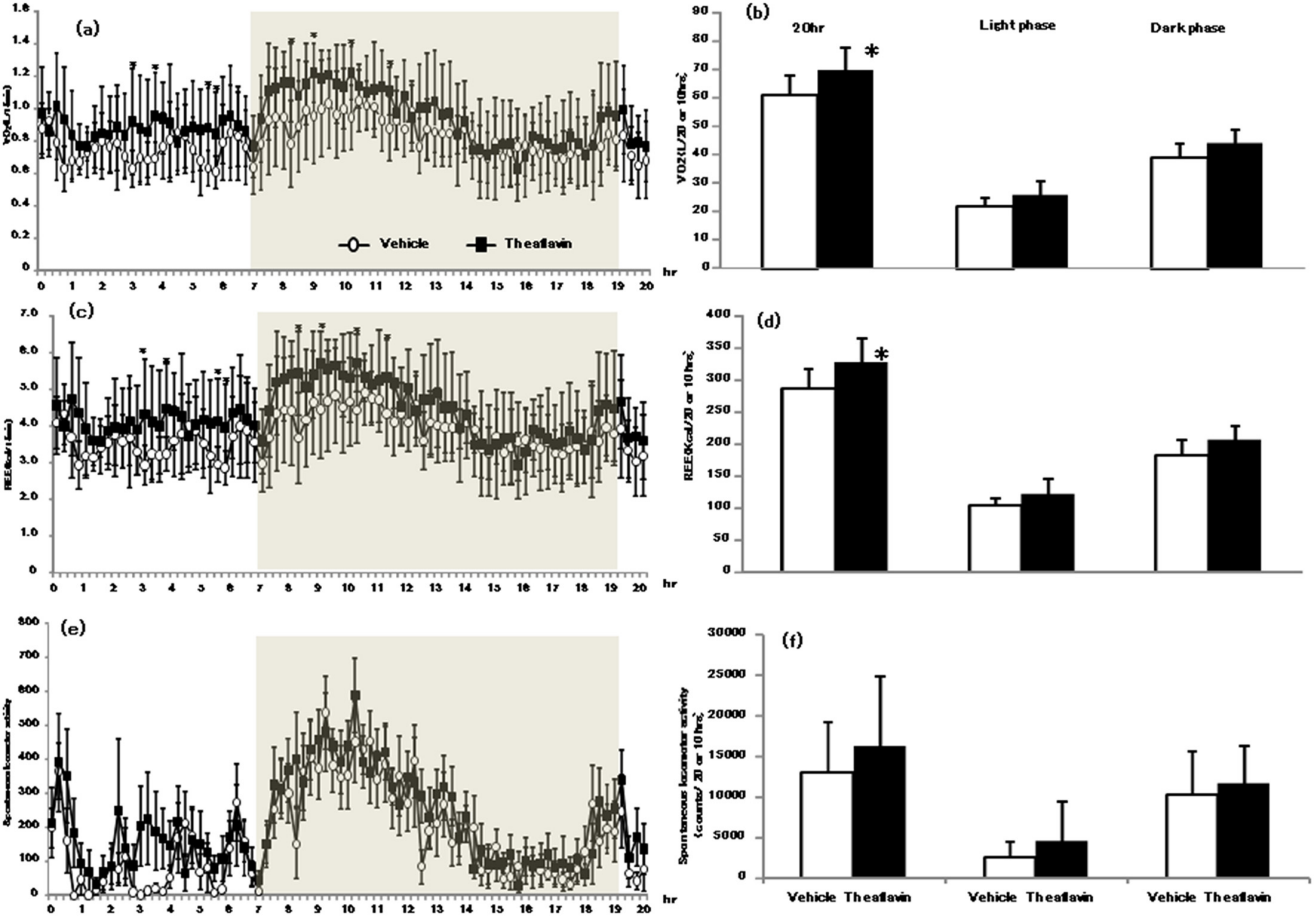
Oxygen consumption (VO_2)_ and energy expenditure (EE) 20 hr after administration of theaflavin. The respiratory exchange ratio (RER) was calculated using VO_2_ and carbon dioxide excretion (VCO_2_) and the Weir equation. Total VO_2_ and EE during the light (12:00–18:00 and 6:00–8:00) or dark (18:00–6:00) cycles are shown in b and d. The locomotor activity of the animals 20 hr after administration of vehicle or theaflavin is shown in e, while locomotor activity of the mice during the total, light or dark cycles is shown in f. The mice were administrated either vehicle (n = 8) or 10 mg/kg theaflavin (n = 8). The values represent the mean ± standard deviation. The statistical analyses were performed two way ANCOVA (a,c,e) post hoc comparisons with the vehicle group were made by the two-tailed followed by Dunnett's test. Significantly different from vehicle, *p<0.05.

The VO_2_ and EE results during the measurement period are shown in Fig 1a and 1c. VO_2_ was was marginally higher during the measurement period in the TF group compared with the vehicle group (Fig 1a). A similar trend was observed for the EE results (Fig 1c). The total VO_2_ and EE measured during the total and dark (18:00–6:00) or light (12:00–18:00 and 6:00–8:00) cycles are shown in Fig 1b and 1d. Total VO_2_ and EE were significantly higher in the TF group compared with the vehicle group. There were no significant changes in locomotor activity (Fig 1e and 1f) and RER (S1 Fig) in the experimental groups throughout the measurement period.

### UCPs and PGC-1α mRNA levels in BAT and the gastrocnemius muscle

**Fig 2:**
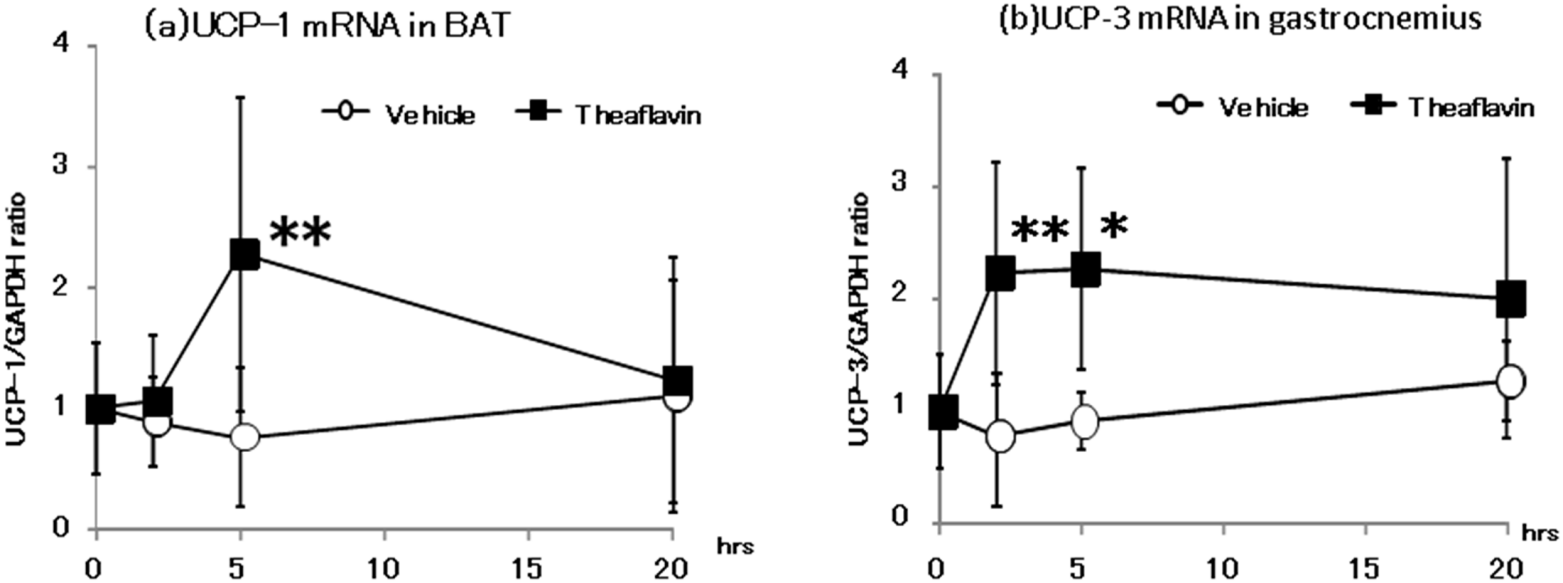
Expression of mRNA for UCP-1 in BAT (a) and UCP-3 in gastrocnemius muscle (b) after administration of vehicle or theaflavin. The animals were euthanized before and 2, 5, and 20 hr after administration of vehicle or 10 mg/kg theaflavin (n = 8). The values represent the mean ± standard deviation. The statistical analyses were performed two way ANCOVA post hoc comparisons with the vehicle group were made by the two-tailed followed by Dunnett's test. Significantly different from vehicle, *p<0.05, **p<0.01.

**Fig 3:**
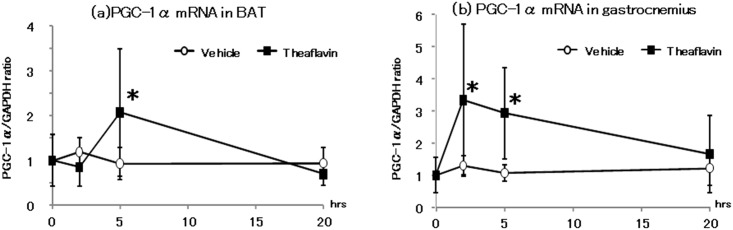
Expression of mRNA for PGC1-α in BAT (a) or gastrocnemius (b) after administration of vehicle or theaflavin. The animals were euthanized before and 2, 5, and 20 hr after administration of vehicle (n = 8) or 10 mg/kg theaflavin (n = 8). The values represent the mean ± standard deviation. The statistical analyses were performed two way ANCOVA, post hoc comparisons with the vehicle group were made by the two-tailed followed by Dunnett's test. Significantly different from vehicle, *p<0.05.

The change in mRNA expression of UCP-1 in BAT is shown in Fig 2a. The levels of UCP-1 mRNA increased significantly 2-hr after administration of TF compared with that of the vehicle. mRNA expression of UCP-3 in the gastrocnemius muscle was also increased significantly 2 and 5-hr after ingestion of TF (Fig 2b). The levels of PGC-1α mRNA in BAT and the gastrocnemius muscle are shown in Fig 3a and 3b. Compared with mice in the vehicle group, administration of TF caused a significant increase in expression of PGC-1α mRNA in BAT after 2-hr, and in the gastrocnemius muscle after 2 and 5-hr.

### Phosphorylation of AMPK1α in BAT and the gastrocnemius)

**Fig 4:**
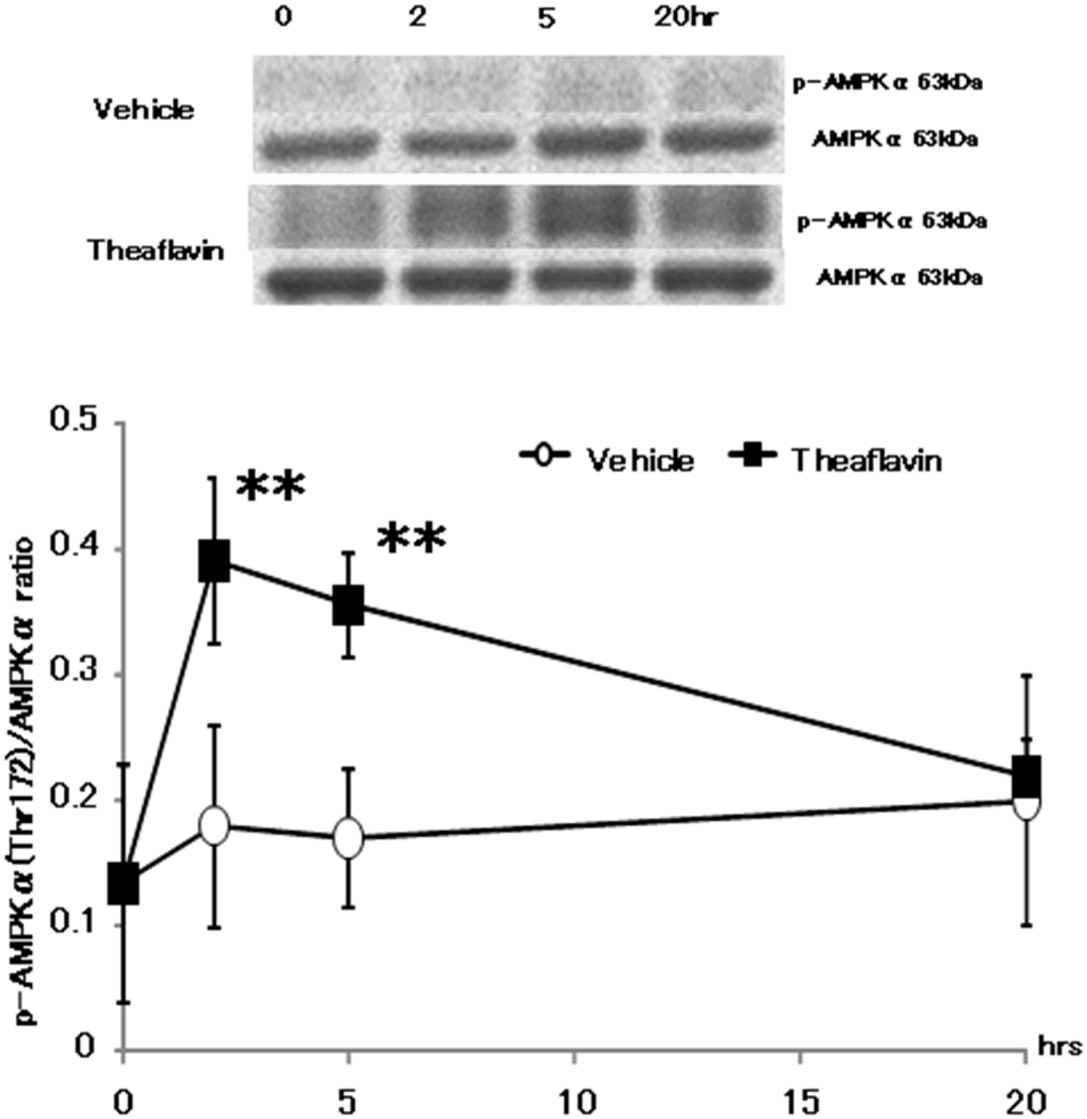
Phosphorylation of AMPK1α in gastrocnemius muscle after administration of vehicle or theaflavin. The animals was euthanized before and 2, 5, and 20 hr after administration of the vehicle (n = 8) or 10 mg/kg theaflavin (n = 8). The values represent the mean ± standard deviation. The statistical analyses were performed two way ANCOVA, post hoc comparisons with the vehicle group were made by the two-tailed followed by Dunnett's test. Significantly different from vehicle, **p<0.01.

As shown in Fig 4 (see also S2a and S2b Fig) phosphorylation of AMPK1α in the gastrocnemius muscle was increased 2 and 5-hr after treatment with TF compared with the vehicle group, with the level of phosphorylated AMPK1α returning to normal 20-hr after ingestion.

## Discussion

The present study showed that a single oral administration of 10 mg/kg TF increased oxygen consumption and energy expenditure for approximately 12-hr without an increase in locomotive activity, compared with values measured following administration of distilled water (Fig 1). We also observed a significant increase in mRNA expression of UCPs and PGC-1α 2 and 5-hr after the mice had received TF (Fig 2). In addition, phosphorylation of AMPK1α in the gastrocnemius muscle was increased following treatment with TF (Fig 3). These results suggested that TF stimulate metabolic activity via induction of UCPs and PGC-1α, which are partlial responsible for adaptive thermogenesis and lipolysis.

As described above, consumption of black tea is associated with significant primary prevention of cardiovascular diseases, by reducing plasma LDL cholesterol levels and blood pressure [7, 8]. Black tea contains not only theaflavins but also green tea catechins and caffeine [12]. As suggested by previous meta-analyses, it remains unclear which component in black tea contributes to the risk reduction of cardiovascular disease. In the present study, we found that a theaflavin mixture caused increased energy expenditure, accompanied by an increase in UCP and PGC-1α mRNA expression and phosphorylated AMPKα in BAT and the gastrocnemius muscle in the absence of green tea catechins and caffeine. Loke et al. also reported that ingestion of TF significantly attenuated the size of atherosclerotic lesions in the aortic sinus and thoracic aorta in apolipoprotein E knockout mice [14]. It has also been reported that 12-wk supplementation of theaflavin-enriched tea extract caused a significant decrease in plasma total and LDL cholesterol levels and triglyceride levels, and increased HDL-cholesterol in subjects with mild to moderate hypercholesterolemia [15]. It was also reported that theaflavin-rich fermented tea decreased body and adipose tissue weight, and plasma triglyceride levels in rats [16]. The ingestion of theaflavin-rich tea also reduced serum glucose levels and hepatic triglyceride concentrations and increased fecal fat excretion in mammals [17]. These anti-dyslipidemia effects of theaflavins are due to a reduction in incorporation of triglyceride [16] and cholesterol [18] into micelles. The results of the current study suggest that the risk reduction activity of theaflavin on cardiovascular disease possibly contributes to other metabolic modifications. There have been reported a single dose of polyphenol enhancement of energy expenditure [19], increase insulin response [20], elevation of AMPK phosphorylation [21]. These data supported that one or more mechanisms are presented in metabolic effect of polyphenol other than enhancement of lipid excretion. Further study was needed to explain the contribution of theaflavin on the risk reduction of cardiovascular disease by the ingestion of black tea.

The bioavailability of theaflavin after ingestion of black tea is also poorly understood. Although several methods have been developed for measuring theaflavin in blood or tissues, it remains difficult to determine blood theaflavin concentrations after consumption of black tea [22,23]. These studies suggested that theaflavin had relatively low bioavailability compared with other polyphenols.

Recently, we showed procyanidins, a type of catechin oligomer, had similar properties to theaflavin, and whilst having low bioavailability, also enhanced energy expenditure and increased expression of genes responsible for thermogenesis and lipolysis [24]. Procyanidins have also been shown to increase blood adrenaline concentration 2-hr after ingestion. These results suggested that catecholamines are involved in the metabolic changes induced by procyanidin It is well established that the sympathetic nerve system (SNS) and the catecholamine, adrenaline and noradrenaline, which are neurotransmitters in the SNS, play important roles in the regulation of energy expenditure [25,26]. UCP-1 in BAT upregulation is known the resulting β3 adrenergic recptor stimulation [27], PGC-1α upregulation and AMPK 1 phosphorylation in skeletal muscle were also known to through β2 adrenergic action [28], by which catecholamine excreted from the end of sympathetic nerve. However, further experiments are required to determine whether catecholamine dynamics contribute to the metabolic modifications caused by of theaflavins.

In conclusion, we found that a single dose of theaflavin rich fraction enhanced energy expenditure accompanied by an increase in expression of metabolic genes. These findings may contribute to the primary risk reduction of cardiovascular diseases associated with consumption of black tea, as suggested by previous epidemiological studies.

## Supporting Information

S1 Fig Carbon oxide excretion(VCO_2)_ 20 hr after administration of theaflavin. Total VCO_2_ during the light (12:00–18:00 and 6:00–8:00) or dark (18:00–6:00) cycles are shown. The mice were administrated either vehicle (n = 8) or 10 mg/kg theaflavin (n = 8). The values represent the mean ± standard deviation. The statistical analyses were performed two way ANCOVA (a) post hoc comparisons with the vehicle group were made by the two-tailed followed by Dunnett's test. Significantly different from vehicle, *p<0.05.
(TIF)

S2 Fig Phosphorylation of AMPK1α in gastrocnemius muscle after administration of vehicle or theaflavin. (Western blot analysis of protein extracts from 0, 2, 5 or 20 hours in gastrocnemius muscle either vehicle (a) or treated with theaflavin (b). Full-length blots to Fig 4 of AMPK and phosphorylated AMPK are shown. Protein samples were run under the same experimental conditions.
(PDF)

S1 Table Primers used for Real-Time Quantitative Reverse-Transcriptase Polymerase Chain Reaction. (TIF)
