# Supplementary figures and images for: A Single Oral Administration of Theaflavins Increases Energy Expenditure and the Expression of Metabolic Genes
Source: PLoS One. 2015 Sep 16;10(9):e0137809. doi: 10.1371/journal.pone.0137809 (PMC4574049; doi:10.1371/journal.pone.0137809)

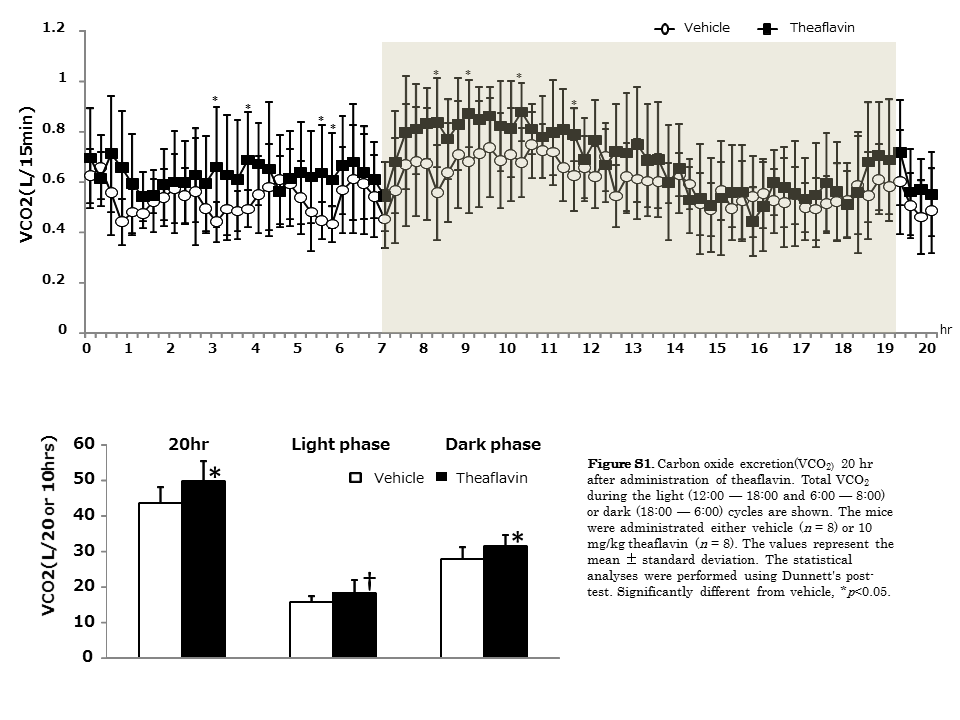

Supplement: S1 Fig — Total VCO2 during the light (12:00–18:00 and 6:00–8:00) or dark (18:00–6:00) cycles are shown. The mice were administrated either vehicle (n = 8) or 10 mg/kg theaflavin (n = 8). The values represent the mean ± standard deviation. The statistical analyses were performed two way ANCOVA (a) post hoc comparisons with the vehicle group were made by the two-tailed followed by Dunnett's test. Significantly different from vehicle, *p<0.05. (TIF) [file pone.0137809.s001.TIF]

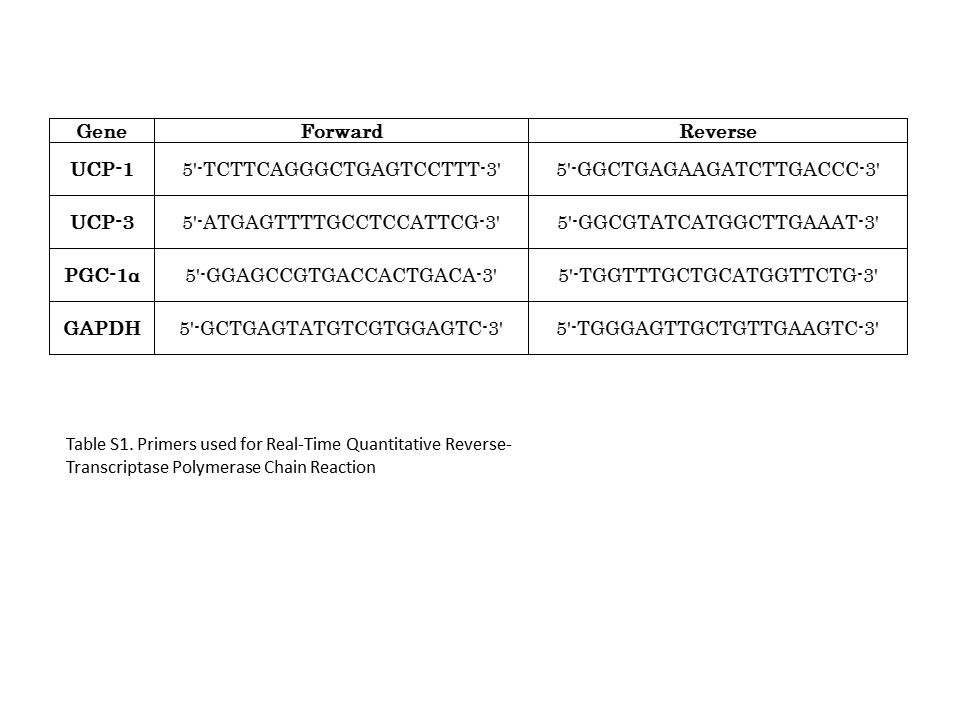

Supplement: S1 Table — (TIF) [file pone.0137809.s003.TIF]
