# Supplementary material for: A Single Oral Administration of Theaflavins Increases Energy Expenditure and the Expression of Metabolic Genes
Source: PLoS One. 2015 Sep 16;10(9):e0137809. doi: 10.1371/journal.pone.0137809 (PMC4574049; doi:10.1371/journal.pone.0137809)
Supplement: S2 Fig — (Western blot analysis of protein extracts from 0, 2, 5 or 20 hours in gastrocnemius muscle either vehicle (a) or treated with theaflavin (b). Full-length blots to Fig 4 of AMPK and phosphorylated AMPK are shown. Protein samples were run under the same experimental conditions. (PDF) [file pone.0137809.s002.pdf]

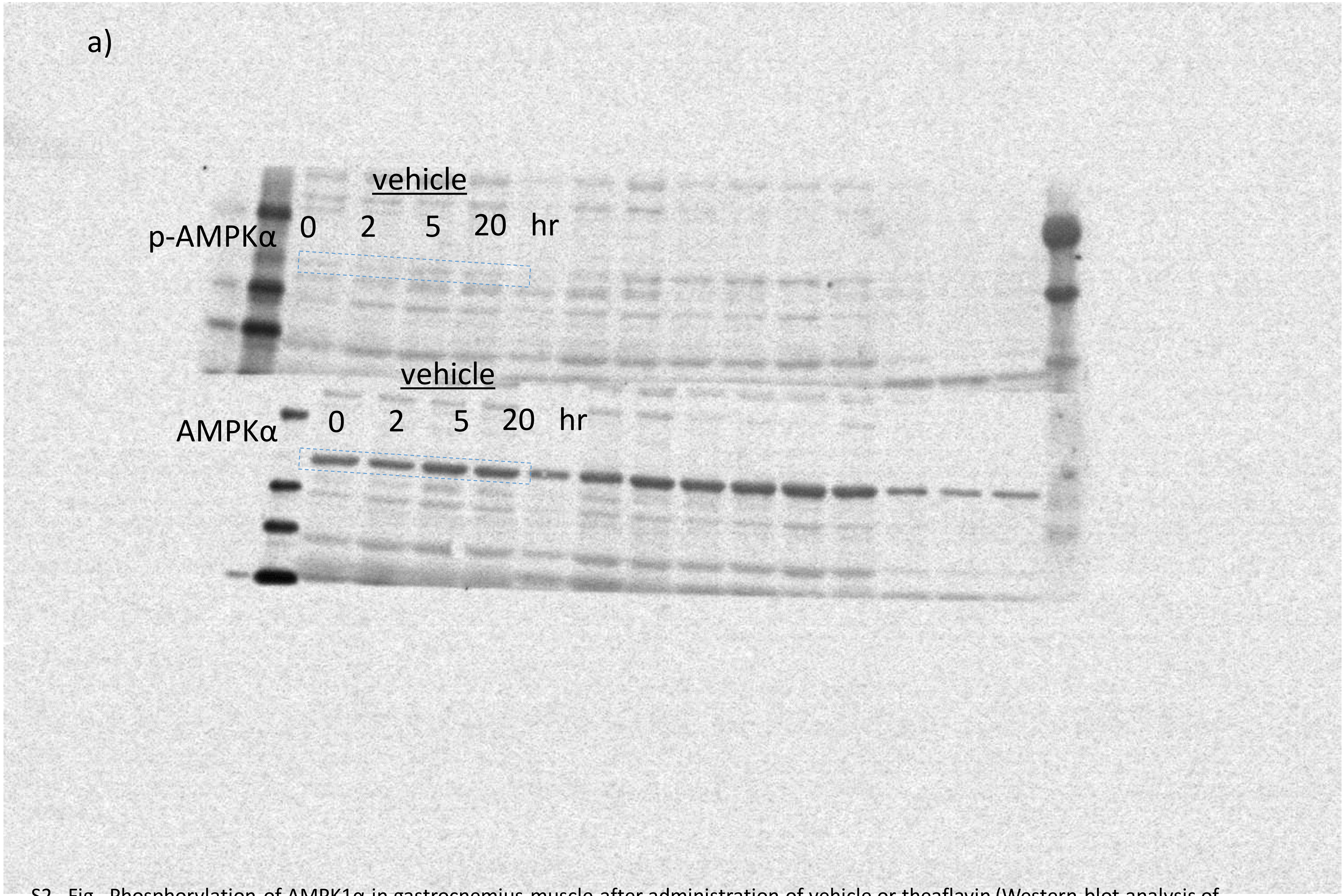

S2 Fig.. Phosphorylation of AMPK1α in gastrocnemius muscle after administration of vehicle or theaflavin.(Western blot analysis of protein extracts from 0, 2, 5 or 20 hours in gastrocnemius muscle either vehicle (a) or treated with theaflavin (b). Full-length blots to Figure 4 of AMPK and phosphorylated AMPK are shown. Protein samples were run under the same experimental conditions.

S2a Fig

b)

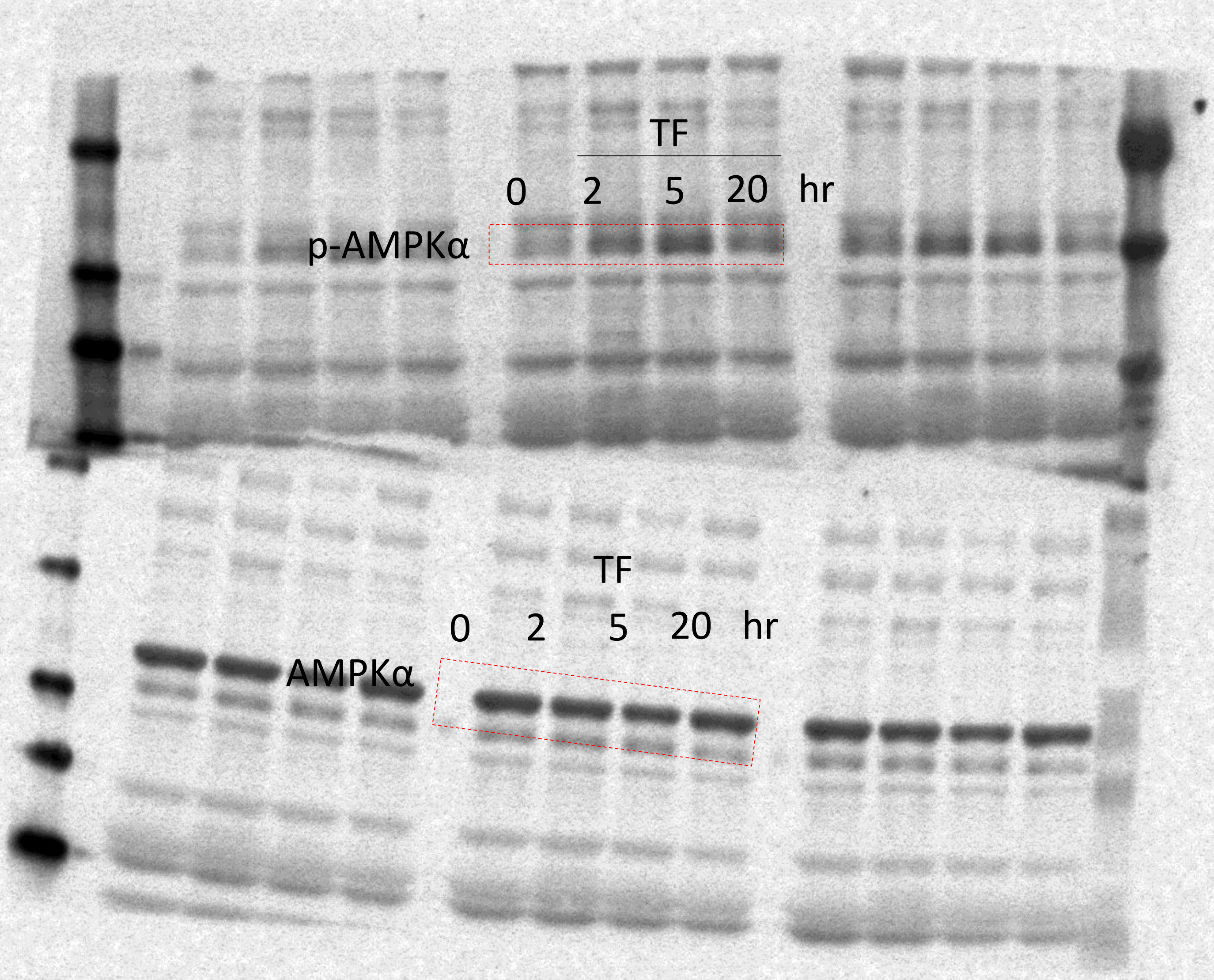

S2b Fig.. Phosphorylation of AMPK1α in gastrocnemius muscle after administration of vehicle or theaflavin.(Western blot analysis of protein extracts from 0, 2, 5 or 20 hours in gastrocnemius muscle either vehicle (a) or treated with theaflavin (b). Full-length blots to Figure 4 of AMPK and phosphorylated AMPK are shown. Protein samples were run under the same experimental conditions.

S2b Fig
